# Food protein-induced enterocolitis syndrome in an infant triggered by prunes

**DOI:** 10.1186/s13223-023-00787-2

**Published:** 2023-04-23

**Authors:** Uliana Kovaltchouk, Thomas Gerstner

**Affiliations:** 1 Division of Clinical Immunology and Allergy, Department of Medicine, Western University, London, Ontario Canada; 2 Section of Allergy and Clinical Immunology, Department of Pediatrics and Child Health, University of Manitoba, Winnipeg, Manitoba Canada

## Abstract

**Background:**

Food protein-induced enterocolitis syndrome (FPIES) is a non-IgE mediated food allergy that has a cumulative incidence of 0.015 to 0.7% in infants [1]. The most common allergens causing FPIES reactions include cow’s milk, followed by soy, grains, and rice [1, 3]. Increasing clinical awareness of FPIES has resulted in the expansion of emerging triggers of FPIES, including fruit antigens.

**Case presentation:**

We describe an infant diagnosed with FPIES to prune.

**Conclusion:**

Fruit allergens are an emerging group of triggers for FPIES, both in their fresh and dried forms. To our knowledge, this case is the first presentation of FPIES to prunes (dehydrated plum). This case highlights that careful history taking can avoid unnecessary investigations and delay in diagnosing FPIES.

## Background

Food protein-induced enterocolitis syndrome (FPIES) is a non-IgE mediated food allergy that has a cumulative incidence of 0.015 to 0.7% in infants [1]. It is caused by a reaction against food proteins in the gut that results in projectile, repetitive emesis and diarrhea [1, 2]. The most common allergens causing FPIES reactions include cow’s milk, followed by soy, grains, and rice [1, 3]. Increasing clinical awareness of FPIES has resulted in the expansion of emerging triggers of FPIES. Herein we present a case of prune induced FPIES in an infant.

## Case presentation

A seven month old, exclusively breast fed female infant, presented to the Allergy and Immunology clinic with a history of repetitive projectile emesis after consuming prune puree at four and a half months of age. She was born after full term gestation from spontaneous vaginal birth after an uncomplicated pregnancy, with a birth weight of 3.253 kg. Before presenting to the Clinical Immunology and Allergy clinic, she was diagnosed with idiopathic epilepsy at 3 months of age, and was started on phenobarbital 5 mg/ml with complete resolution of her seizures. She began complimentary feeding with wheat based cereal at four months of age with good tolerance. She was subsequently introduced to various fruit purees, including pear, strawberry, banana, peaches, orange, and apple with good tolerance. She was given prune puree mixed with wheat cereal, and within 2 h, had repetitive projectile emesis. There was no diarrhea, nor any symptoms consistent with IgE mediated food allergy. No other specific triggers were identified. Following emesis the infant displayed signs of lethargy for 2 h, however, recovered at home without any medical intervention. She was given prune puree again 2 weeks later with no other foods, and developed repetitive, projectile emesis. No history of allergic rhinoconjunctivitis, or asthma was identified. The infant had a background history of mild atopic dermatitis, more frequently seen in young children with FPIES. Investigations revealed negative skin prick testing to prune puree. On the basis of her history, a clinical diagnosis of FPIES to prunes was made, and the infant had no further episodes of vomiting strictly avoiding prunes and plums in their diet. She has not developed FPIES or IgE mediated hypersensitivity to any other foods. After a patient centered discussion, the decision was made to strictly avoid plums and prunes, and to challenge the infant to prunes when she reaches 3 years of age.

## Discussion

Fruit is a very uncommon cause of FPIES, with banana being the most commonly implicated fruit in the literature, summarized in Table 1 [4–13]. To our knowledge, prune has never been previously described to cause FPIES in infants or adults. There are two previous documented case of FPIES to plum, however, both clinical presentations were not extensively reviewed, but, rather extrapolated retrospectively [9, 12]. Although exceedingly rare, FPIES to fruits should be included in the differential for an infant that presents with vomiting, diarrhea and subsequent symptoms of dehydration in addition to gastroenteritis, anaphylaxis, methemoglobinemia, sepsis, and metabolic diseases. This is important to avoid unnecessary investigations, mismanagement, and ultimately delay in diagnosing FPIES. Many features of FPIES are still being defined, including the antigens responsible for eliciting reactions. It has been speculated that lipid transfer proteins could be responsible for fruit-induced FPIES [4]. Our patient was able to tolerate other fruits belonging to the Rosaceae family, including apples, peaches, pear, and strawberries suggesting her reaction to prunes was to an antigen not homologous among the family. Prunes have previously been identified as a lower risk food for eliciting a FPIES reaction [14]. Based on all previous epidemiological data available this remains true, however, an index of suspicion should remain high for any food that has a clinical history in keeping with causing FPIES, regardless of previous classification.

**Table 1:** Summary of fruits that cause food protein-induced enterocolitis reaction from previous literature

Study	Number of patients and implicated fruit
Bruni et al. 2008 [4]	1 patient: Fruit mix (banana, pear, apple)
Mehr et a. 2009 [5]	1 patient: Banana
Sopo et al. 2012 [6]	2 patients : Banana
Federly et al. 2013 [7]	1 patient: Orange juice
Don et al. 2013 [8]	1 patient: Banana
Ruffner et al. 2013 [9]	Reported percentages ~ 3.5 patients: Banana < 1% patients: Plum < 1% patients: Peach < 1% patients: Strawberry < 1% patients: Watermelon < 1% patients: Avocado
Fiocchi et al. 2014 [10]	1 patient: Banana/apple
Morena et al. 2018 [11]	1 patient: Banana 1 patient: Banana/apple
Blackman et al. 2019 [12]	18 patients: Banana 12 patients: Avocado 8 patients: Apple 2 patients: Blueberry 2 patients: Mango 1 patient: Peach 1 patient: Strawberry 1 patient: Plum
Maciag et al. 2020 [13]	57 patients: Avocado 53 patients: Banana 35 patients: Apple 22 patients: Coconut 19 patients: Pear 19 patients: Blueberry 12 patients: Strawberry 11 patients: Peach 19 patients: Pear 9 patients: Mango 8 patients: Orange 8 patients: Watermelon 7 patients: Raspberry 7 patients: Grape 6 patients: Pineapple 5 patients: Kiwi 3 patients: Cherry 1 patient: Honeydew 1 patient: Nectarine

Consensus guidelines suggest that treatment of patients with FPIES should include eliminating the suspected allergen, and providing medical treatment should there be an accidental exposure [3]. Additionally, when to re-challenge infants, and the procedure for up-dosing the culprit food that results in FPIES, particularly with less implicated foods such as prunes, has not been clearly elucidated. Generally, oral food challenges to determine resolution have been attempted approximately 12–18 months after the initial reaction, often in conjunction with shared decision making with patients [3].

## Conclusion

In summary, fruit allergens are an emerging group of triggers for FPIES, both in their fresh and dried forms. To our knowledge, this case is the first presentation of FPIES to prunes (dehydrated plum). This case highlights that careful history taking can avoid unnecessary investigations and delay in diagnosing FPIES.
